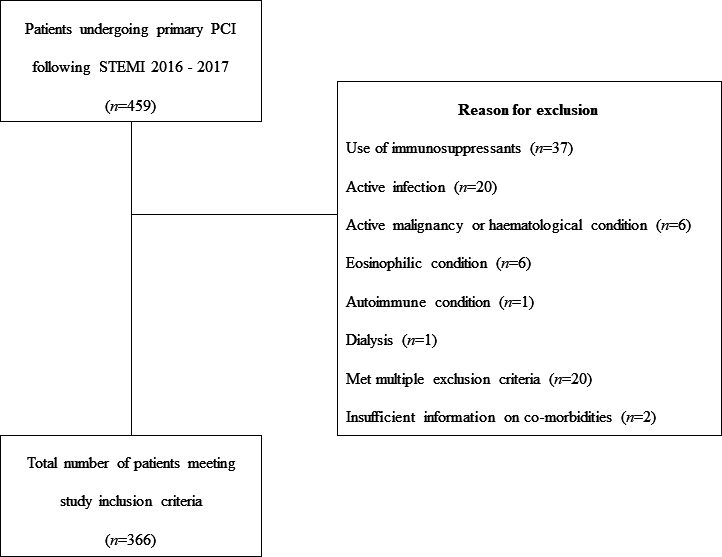# Using eosinophil response to predict cardiovascular outcomes in patients with ST- elevation myocardial infarction who undergo primary percutaneous coronary intervention

**DOI:** 10.1016/j.ijcrp.2025.200383

**Published:** 2025-03-06

**Authors:** Joyce Lim, Trent Williams, Lucy Murtha, Nishani Mabotuwana, Conagh Kelly, Doan Ngo, Andrew Boyle

**Affiliations:** a Heart and Stroke Research Program, Hunter Medical Research Institute, New Lambton Heights, NSW, Australia; b College of Health, Medicine and Wellbeing, University of Newcastle, Callaghan, NSW, Australia; c Department of Cardiology, John Hunter Hospital, New Lambton Heights, NSW, Australia

**Keywords:** Eosinophils, ST-Elevation myocardial infarction, Primary percutaneous coronary intervention, Biomarker, Major adverse cardiovascular events

## Abstract

**Objective:**

Eosinophils have been implicated in mediating the inflammatory response after ST-elevation myocardial infarction (STEMI), but its role as a biomarker predicting major adverse cardiovascular events (MACE) remains unclear. We aimed to evaluate the predictive value of eosinophil response on 30-day and 1-year MACE post primary percutaneous coronary intervention (PCI) after STEMI.

**Methods:**

Single centre retrospective cohort study of STEMI patients undergoing PCI. Eosinophil response was defined as the change in peripherally circulating eosinophils cell count at admission minus 48 h post primary PCI. Primary endpoints were 30-day and 1-year MACE. Receiver operating characteristic (ROC) curves were created to identify optimal cut-off predicting MACE. Multivariate logistic regression analyses were used to determine if the ROC cut-off was an independent predictor of MACE.

**Results:**

Of the 366 patients in this study (median age 61 years [53.0–71.0]; 267 males [73 %]), 41 patients (11.2 %) and 78 patients (21.3 %) developed MACE at 30-days and 1-year. The optimal ROC curve cut-off predicting MACE was an eosinophil response of greater than −0.05 × 10^9/L (ΔEos > −0.05). It had a sensitivity, specificity, and positive and negative predictive value of 83, 39, 6 and 98 % for 30-day MACE, and 74, 39, 19 and 88 % for 1-year MACE. An ΔEos > −0.05 change was associated with a threefold higher likelihood of MACE at 30-days (*OR* 3.1*, 95 % CI* 1.04–9.07*, p=*0.042), but not 1-year

**Conclusion:**

An eosinophil response of −0.05 × 10^9L at 48 h following primary PCI post STEMI is highly sensitive at predicting 30-day MACE, and in its absence, holds a high negative predictive value.

## Highlights

• **What is already known about this topic**
• A decrease in \ eosinophils post STEMI is predictive of higher in-hospital mortality and morbidity, but their on longer term adverse cardiovascular outcomes remains unknown.
• **What this study adds**
• This study evaluates whether eosinophil response post primary PCI for STEMI has any predictive value on 30-day and 1-year MACE and mortality.
• **How this study might affect clinical practice**
• The results of this study may provide clinician a simple and useful biomarker to help risk stratify patients and evaluate treatment response post primary PCI following a STEMI.

## Graphical abstract

**Figure:**
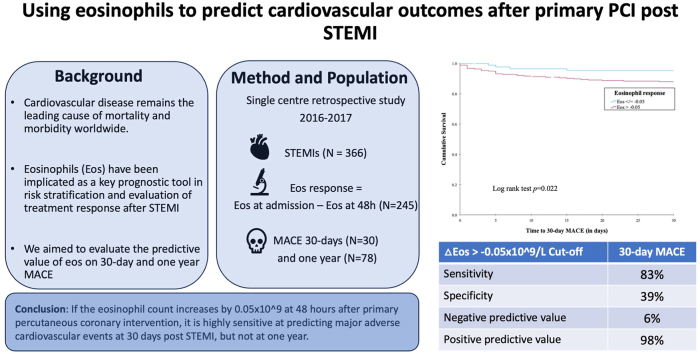

## 1 Background

Despite advances in evidence-based interventions and therapeutics for cardiovascular disease, it remains a leading cause of mortality and morbidity worldwide [1]. Of the cardiovascular diseases, myocardial infarctions (MI) is the principle driver of cardiovascular deaths [2]. As a consequence, there has been an intense focus on the identification and development of novel biomarkers as a prognostic tool for risk stratification and evaluation of treatment response. In particular, there is growing interest in the predictive value of eosinophils following ST-elevation myocardial infarction (STEMI).

Eosinophils are granulocytes that represents less than 5 % of the leukocyte population, and play a pivotal role in allergic conditions and helminth infections [3]. However more recently, they have been implicated in mediating the inflammatory response following myocardial infarction [4]. Decreased eosinophils have been observed in patients following STEMIs and have been associated with more severe coronary artery disease [[5], [6], [7]].

As a biomarker for major adverse cardiovascular events (MACE), their predictive value remains less well-defined. The predictive value of eosinophil ratios has been increasingly studied as a novel biomarker. For example, both lower eosinophil to lymphocyte ratio and eosinophil to monocyte ratio following STEMI have been linked with increased in-hospital mortality and long-term MACE [[8], [9], [10]]. However, using these ratios, it remains unclear whether the observed increase in short and long-term morbidity and mortality are due to a decrease in eosinophil or an increase in the lymphocyte or monocyte component.

In isolation, a decrease in the peripherally circulating eosinophil post STEMI has been shown to be predictive of higher in-hospital mortality and MACE [11,12]. However, to date, no study has evaluated the predictive value of eosinophils in STEMI on longer term mortality and morbidity rates. In this study, we therefore aim to evaluate whether eosinophil response post primary percutaneous coronary intervention (PCI) for STEMI has any prognostic value in 30-day and 1-year MACE and mortality.

## 2 Method

### 2.1 Study design

Single centre retrospective cohort study of all STEMI patients undergoing primary PCI at John Hunter Hospital between 2016 and 2017. All patients admitted with a STEMI were eligible for inclusion into the study. Patient care was per the National recommended guidelines [13], at the treating physician's discretion. The study was reported in line with the Strengthening the Reporting of Observational Studies in Epidemiology (STROBE) guidelines [14] and approved by the Hunter New England Research Ethics Office.

Ethics approval to conduct this study was granted by Hunter New England Human Research Ethics Committee (HNE HREC) - Approval No. 2023/ETH01757. This study complies with the Declaration of Helsinki. Approval to waive patient consent for this study was sought and approved by the HNE HREC.

### 2.2 Study population

The inclusion criteria for the study were patients aged 18 years or older presenting with two confirmed STEMIs, as defined by: [1] chest discomfort; [2] 1 mm ST elevation in at least 2 contiguous limb leads, 2 mm in the chest leads or a new left bundle branch block; and [3] troponin greater than the 99th centile upper limit of normal [15]. All patients were required to undergo primary PCI.

Exclusion criteria included: [1] eosinophilic related conditions; [2] active infections; [3] malignancy or haematological condition; [4] inflammatory or autoimmune conditions; [5] dialysis patients; and [6] use of immunosuppressants.

### 2.3 Study variables

#### 2.3.1 Baseline demographics

Baseline demographic information was collected from electronic medical records. Extracted information included age, gender, body mass index, co-morbidities, medication history and cardiac risk factors.

#### 2.3.2 Infarct characteristics

Infarct characteristics of interest included clinical, echocardiographic and angiographic features. Clinical features included door to balloon time, peak troponin and length of hospital stay. The degree of left ventricular systolic dysfunction was characterised using transthoracic echocardiogram. Angiographic characteristics included the culprit vessel, TIMI flow post PCI and the presence of multi-vessel coronary artery disease. Where available, information was extracted from the electronic medical records, and verified by reviewing the echocardiographic and angiographic images.

#### 2.3.3 Outcome measures

Eosinophil response was defined as the change in peripherally circulating eosinophils cell count at admission minus 48 h post STEMI. Full blood counts were taken within 24 h of admission and at daily intervals post PCI. Other biomarkers obtained included baseline serum creatinine and high sensitivity troponin I.

The primary outcome measured was any major adverse cardiovascular events (MACE), as defined by all-cause mortality, new heart failure, recurrent acute coronary syndrome, any cardiac related admission or non-fatal strokes, at 30-days or 1-year. The secondary outcome was any all-cause mortality at 30-days or 1-year. Outcome data were obtained through the institutional cardiac and stroke outcome unit database, which has previously been described [16]. This database prospectively records all cardiac and stroke admissions to public hospitals within the area health service based on discharge ICD codes.

### 2.4 Statistical analysis

Continuous variables were expressed as mean (standard deviations, *SD*) or median (interquartile range, *IQR*), and categorical variables as an absolute value (percentage, %). Normality was tested with the Shapiro-Wilks test. Statistical differences in the baseline, infarct and outcome variables were tested using Independent Samples T-test for parametric continuous data, Kruskall Wallis H test for non-parametric continuous data and Pearson Chi-Square test for dichotomous variables. Receiver operating characteristic (ROC) curves were created to identify the optimal cut-off predicting MACE using the eosinophils response at 48 h. Patients with missing eosinophil response were excluded from the analysis. Kaplan-Meier estimates were used to summarise incidence of 30-day and 1-year MACE and mortality, and compared using the Log-Rank test. Univariate and multivariate logistic regression analyses were conducted to determine if the ROC curve cut-off was an independent predictor of the primary and secondary endpoint.

Significant tests were 2-sided, and deemed statistically significant when *p-*value<0.05. All analyses were carried out using Excel version 16.54 (Microsoft Corporation, USA) and SPSS for Windows version 28.0 (SPSS Inc., Chicago, IL, USA).

## 3 Results

### 3.1 Baseline characteristics

A total of 366 patients met the inclusion criteria for the study (median age 61 years [53.0–71.0]; 267 males [73 %] (Supplement Fig. 1). All patients were followed up for a total of 12 months. Forty-one patients (11.2 %) developed MACE by 30 days and 78 patients (21.3 %) by one year. The all-cause mortality rate was 4.6 % and 6.3 %, respectively. No significant differences in 1-year MACE were observed, except for age, LAD culprit lesions and LVEF (Table 1).

**Table 1:** Demographics, medical comorbidities and medication use, haematological parameters at admission and infarct characteristics of patients according to 1 year MACE. *Abbreviations*: BMI, body mass index; MI, myocardial infarction; HTN, hypertension; DM, diabetes mellitus; ACE, angiotensin converting enzyme inhibitors; BB, beta-blockers; Hb, haemoglobin; WCC, white cell count; Cr, Creatinine; DTBT, Door to balloon time; LAD, left anterior descending artery; TIMI, thrombolysis in myocardial infarction; LVEF, left ventricular ejection fraction.

	no MACE (n = 288)	1y MACE (n = 78)	*p*-value
**Baseline Demographics**			
Age, median (IQR), years	60.0 (53.0–68.8)	66.5 (55.5–80.0)	0.005
Male sex, n (%)	214 (74.3)	53 (67.9)	0.262
BMI, median (IQR), kg/m2	29.0 (27.0–32.0)	29.5 (26.9–31.7)	0.898
**Medical comorbidities**			
Previous MI, n (%)	40 (13.9)	15 (19.2)	0.242
HTN, n (%)	147 (51.0)	41 (52.6)	0.811
Dyslipidaemia, n (%)	89 (30.9)	36 (46.2)	0.017
Smoker, n (%)	126 (43.8)	27 (34.6)	0.147
DM, n (%)	54 (18.8)	26 (33.3)	0.009
**Medication use**			
ACEI, n (%)	72 (25.0)	21 (26.9)	0.729
Statin, n (%)	57 (19.8)	22 (28.2)	0.109
BB, n (%)	24 (8.3)	11 (14.1)	0.127
Aspirin, n (%)	47 (16.3)	19 (24.4)	0.093
Clopidogrel, n (%)	17 (5.9)	4 (5.1)	0.808
**Haematological parameters**			
Hb, mean (SD), x10^9	139.5 (16.4)	138.0 (20.5)	0.535
WCC, median (IQR), x10^9	10.7 (8.4–13.4)	11.1 (7.9–14.1)	0.861
Neutrophils, median (IQR), x10^9	7.7 (5.8–10.3)	7.7 (5.6–11.0)	0.794
Lymphocytes, median (IQR), x10^9	1.9 (1.3–2.6)	1.9 (1.3–2.6)	0.904
Monocytes, median (IQR), x10^9	0.6 (0.5–0.8)	0.7 (0.5–0.9)	0.146
Eosinophil, median (IQR), x10^9	0.1 (0.0–0.2)	0.1 (0.0–0.2)	0.805
Baseline Cr, median (IQR), mmolL-1	79 (69–97)	85 (72–101)	0.085
**Infarct characteristics**			
DTBT, median (IQR), min	73.5 (54.0–95.0)	82.0 (61.0–94.0)	0.542
Length of stay, median (IQR), days	3 [3,4]	3 [2–5]	0.155
Peak troponin, median (IQR), mmolL-1	32,336 (10,959–71,665)	37,657 (7735–98,576)	0.802
LAD culprit, n (%)	113 (39.2)	42 (53.8)	0.021
Single-vessel disease, n (%)	38 (13.2)	7 (9.0)	0.314
TIMI III flow achieved, n (%)	278 (96.5)	73 (93.6)	0.951
LVEF, median (IQR), %	54 (45–60)	44 (38–54)	0.001

**Fig. 1:**
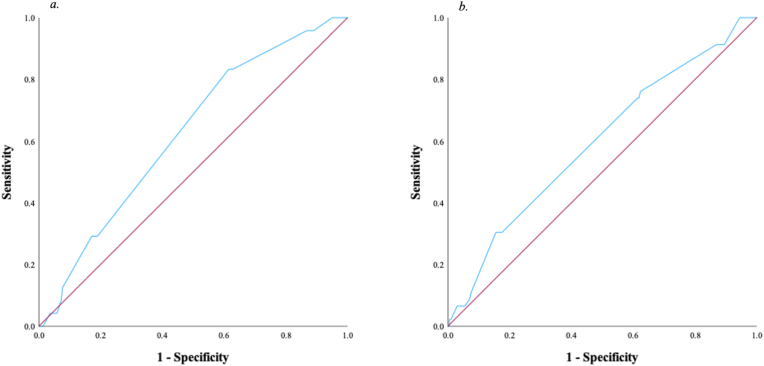
Receiver operating characteristic curves for MACE at *a*. 30 days; *b*. 1 year.

A ROC curve was constructed to identify the best cut-off for eosinophil response at 48 h following a STEMI to predict 30-day and 1-year MACE. Eosinophil response were not recorded for 121 patients (33 %). The best cut-off value was calculated as an eosinophil response of greater than −0.05 × 10^9/L (Fig. 1). For 30-day MACE, it had a sensitivity, specificity, and positive and negative predictive value of 83, 39, 6 and 98 %, respectively, and diagnostic accuracy of 62 %. For 1-year MACE, the cut-off had a diagnostic accuracy of 60 % with a sensitivity, specificity, and positive and negative predictive value of 74, 39, 19 and 88 %, respectively.

Patients were grouped into those with less than or equal to the ROC curve cut-off (ΔEos ≤ −0.05) or greater than the ROC curve cut-off (ΔEos > −0.05). No between group differences were observed between the two ROC curve groups, except in their haematological parameters at admission. The ΔEos > −0.05 group had significantly lower neutrophil counts (7.4 × 10^9/L, *IQR* 5.4–10.2 vs 8.8 × 10^9/L, *IQR* 6.5–11.5; *p* = 0.009) and higher lymphocyte counts (2.2 × 10^9/L, *IQR* 1.3–2.9 vs 1.5 × 10^9/L, *IQR* 1.1–2.0; *p* < 0.001) (Table 2).

**Table 2:** Demographics, medical comorbidities and medication use, haematological parameters at admission and infarct characteristics of patients in the ΔEos ≤ −0.05 vs ΔEos > −0.05 group. *Abbreviations*: BMI, body mass index; MI, myocardial infarction; HTN, hypertension; DM, diabetes mellitus; ACE, angiotensin converting enzyme inhibitors; BB, beta-blockers; Hb, haemoglobin; WCC, white cell count; Cr, Creatinine; DTBT, Door to balloon time; LAD, left anterior descending artery; TIMI, thrombolysis in myocardial infarction; LVEF, left ventricular ejection fraction.

	ΔEos ≤ −0.05 × 10^9 (n = 89)	ΔEos > −0.05 × 10^9 (n = 156)	*p*-value
**Baseline Demographics**			
Age, median (IQR), years	62.0 (54.0–70.0)	61.0 (53.0–71.5)	0.887
Male sex, n (%)	71 (79.8)	107 (68.6)	0.059
BMI, median (IQR), kg/m2	29.3 (26.3–32.0)	28.5 (26.5–30.9)	0.282
**Medical comorbidities**			
Previous MI, n (%)	10 (11.2)	24 (15.4)	0.366
HTN, n (%)	42 (47.2)	85 (54.5)	0.272
Dyslipidaemia, n (%)	27 (30.3)	49 (31.4)	0.861
Smoker, n (%)	40 (44.9)	62 (39.7)	0.427
DM, n (%)	16 (18.0)	36 (23.1)	0.348
**Medication use**			
ACE, n (%)	22 (24.7)	41 (26.3)	0.788
Statin, n (%)	15 (16.9)	34 (21.8)	0.352
BB, n (%)	7 (7.9)	14 (9.0)	0.785
Aspirin, n (%)	15 (16.9)	26 (16.7)	0.995
Clopidogrel, n (%)	5 (5.6)	8 (5.1)	0.878
**Haematological parameters**			
Hb, mean (SD), x10^9	137.9 (17.4)	141.0 (18.8)	0.2.09
WCC, median (IQR), x10^9	11.6 (8.7–13.9)	10.9 (8.5–13.7)	0.550
Neutrophils, median (IQR), x10^9	8.8 (6.5–11.5)	7.4 (5.4–10.2)	0.009
Lymphocytes, median (IQR), x10^9	1.5 (1.1–2.0)	2.2 (1.3–2.9)	<0.001
Monocytes, median (IQR), x10^9	0.6 (0.4–0.9)	0.7 (0.5–0.9)	0.052
Eosinophil, median (IQR), x10^9	0.0 (0.0–0.1)	0.1 (0.1–0.2)	<0.001
Baseline Cr, median (IQR), mmolL-1	82 (69–102)	82 (72–99)	0.862
**Infarct characteristics**			
DTBT, median (IQR), min	75.0 (55.8–95.5)	70.5 (51.0–91.8)	0.152
Length of stay, median (IQR), days	3 [2–4]	3 [3,4]	0.054
Peak troponin, median (IQR), mmolL-1	36,278 (13,362–87,763)	38,781 (13,426–86,274)	0.964
LAD culprit, n (%)	34 (38.2)	69 (44.2)	0.358
Single-vessel disease, n (%)	15 (16.9)	19 (12.2)	0.309
TIMI III flow achieved, n (%)	86 (97.7)	146 (93.6)	0.486
LVEF, median (IQR), %	54 (43–58)	50 (42–59)	0631

### 3.2 Incidence of MACE and mortality using the ROC cut-off

**Fig. 2:**
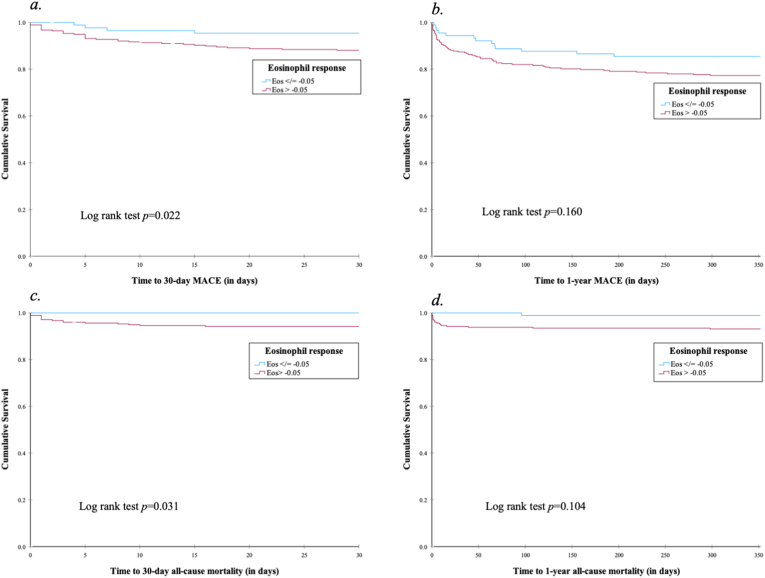
Kaplan meier survival analysis of *a*. 30-day MACE; *b*. 1-Year MACE; *c*. 30-day all-cause mortality and; *d*. 1-Year all-cause mortality.

A higher incidence of 30-day MACE in the ΔEos > −0.05 group was observed (14.1 % vs 4.5 %, *p* = 0.019). However, at 1 year, there was no significant differences in the incidence of MACE (21.8 % vs 14.6 %, *p* = 0.169) (Fig. 2).

Similarly, a higher incidence of all-cause mortality at 30 days was observed in the ΔEos > −0.05 group (5.1 % vs 0.0 %, *p* = 0.030). By 1-year, there was no difference in the incidence of all-cause mortality between the two groups (7.1 % vs 2.2 %, *p* = 0.107) (Fig. 2).

### 3.3 Using the ROC curve cut-off to predict MACE and mortality

In the univariate model with the ROC curve cut-off as the independent variable, it was a significant predictor of 30-day MACE, but not 1-year MACE. When compared to the ΔEos ≤ −0.05 group, the ΔEos > −0.05 group had a significantly increased likelihood of developing 30-day MACE (*OR* 3.5, *95 % CI* 1.16–10.48, *p* = 0.026). The use of the ROC curve cut-off in a multivariate model with other predictors of 30-day MACE, including admission monocyte count and the presence of a LAD culprit, remained a significant independent predictor (*OR* 3.3*, 95 % CI* 1.07–9.99*, p=*0.038) (Supplement Tables 3–4).

In contrast, ROC curve cut-off was not an independent predictor of 1-year MACE, 30-day or 1-year all-cause mortality in the logistic regression analysis (Supplement Table 3). In a univariate analysis, significant predictors of these outcomes included increasing age, medical co-morbidities of dyslipidaemia and diabetes, statin use, elevated neutrophil and lymphocyte count at admission, elevated creatinine levels and LAD culprit disease. However only LAD culprit disease remained a significant predictor of 1-year MACE in the multivariate regression analysis (Supplement Tables 5–7).

## 4 Discussion

This study evaluated the predictive value of eosinophil response on 30-day and 1-year outcomes following STEMIs treated with primary PCI. Our key finding was that eosinophil response is highly sensitive at predicting 30-day and 1-year MACE. After adjusting for other predictors of MACE, the ROC curve cut-off remained an independent predictor of 30-day, but not 1-year MACE. The ROC curve cut-off of −0.05 predicted a threefold higher likelihood of 30-day MACE. This was also reflected in the significantly increased incidence of both 30-day, but not 1-year, MACE and all-cause mortality in the ΔEos > −0.05 group compared to ΔEos ≤ −0.05 group. Interestingly, eosinophil response was also observed to hold a very strong negative predictive value as a biomarker for prognostic stratification.

To date, our study is the first to evaluate the predictive value of eosinophils, independent of other leukocyte components, on longer term morbidity and mortality following primary PCI in STEMI. It is also the first study to highlight the importance of dynamic changes in the eosinophil count following a STEMI in prognosticating cardiovascular outcomes. The results raise two possibilities regarding the eosinophil response that would increase the risk of adverse cardiac outcomes. Either there is an increase in eosinophils at admission relative to baseline which normalises at 48 h post STEMI or there is a decrease in eosinophils at 48 h relative to the eosinophil count at admission.

The former possibility is contrary to current evidence. The current evidence suggests that a low eosinophil count or percentages at admission is a prognostic marker in determining in-hospital mortality, MACE and no-reflow following primary PCI for STEMI [11,12,17]. However, one possible explanation to reconcile these differences, is that peripherally circulating eosinophils may increase following a STEMI before it is sequestered into the infarcted myocardium. Thereby leading to an observed decrease in the eosinophil count in the peripherally circulating blood [6]. In experimental swine models of myocardial infarction, a progressive rise in eosinophils was observed soon after the onset of ischaemia and peaks at 30min, before subsequently returning to pre-infarct levels. In this series of experiments, the decline in the peripherally circulating eosinophil closely parallels these cells infiltrating into the infarcted myocardium for up to 1 month post reperfusion. Significantly higher levels of eosinophils were associated with more extensively damaged myocardium. It is within this paradigm that more severe eosinophilia subsequently observed on the peripherally circulating blood was associated with higher MACE following primary PCI in STEMI [6].

However more recently, the detrimental role of eosinophils in the infarcted myocardium has been challenged. In eosinophil deficient mice, adverse left ventricular remodelling following MI have been observed. More specifically the absence eosinophils was observed to impair the resolution of post infarct inflammation leading to a larger infarct size, greater left ventricular dilatation and poorer left ventricular function [4,18].

In light of such evidence, the role of eosinophils in the pathophysiology of MI remains unclear. However, our data suggests that eosinophils have a predictive role in determining the longer-term prognosis of patients following primary PCI after STEMI. As a biomarker of prognosis, it is clinically valuable as a simple and easily accessible tool to risk stratify patients for therapeutic interventions.

### 4.1 Study limitations

We acknowledge that our study has several limitations. First, as a single-centre retrospective study, it suffers from the usual biases associated with such methodological designs. Second, the admission eosinophil count was taken at different timepoints following the STEMI onset. Our sample population was too small to adjust for time differences in blood sampling, and therefore reduced the power of our study. Third, while our ROC cut-off had a very strong negative predictive value, this was influenced by the relatively low prevalence of 30-day MACE within the study sample and may not be generalisable to populations with higher prevalence of adverse outcomes. Likewise, our study population was limited to STEMI patients undergoing primary PCI and the findings may not apply to the non-PCI population. Last, though the diagnostic accuracy of the ROC cut-off was significant, it has a relatively weak diagnostic accuracy. Potential confounding factors may have been differences in the leukocyte components at admission between our study groups. Future studies are therefore required to address such limitations and to validate our key findings. As a prognostic tool, increased diagnostic accuracy may be achieved by adding eosinophil response to an algorithm of biomarkers to predict 30-day MACE.

## 5 Conclusion

An eosinophil response of −0.05 × 10^9L at 48 h post STEMI, compared to the admission eosinophil count, is highly sensitive at predicting 30-day MACE and mortality following primary PCI, and in its absence, holds a high negative predictive value.

## CRediT authorship contribution statement

**Joyce Lim:** Writing – review & editing, Writing – original draft, Methodology, Formal analysis, Data curation, Conceptualization. **Trent Williams:** Writing – review & editing, Data curation. **Lucy Murtha:** Writing – review & editing, Methodology, Formal analysis. **Nishani Mabotuwana:** Writing – review & editing, Formal analysis. **Conagh Kelly:** Writing – review & editing, Formal analysis, Data curation. **Doan Ngo:** Writing – review & editing, Methodology, Formal analysis. **Andrew Boyle:** Writing – review & editing, Supervision, Methodology, Investigation, Formal analysis, Conceptualization.

## Funding

None.

## Declaration of competing interests

None.

## Figures and Tables

**figs1:**