# Supplementary material for: Using eosinophil response to predict cardiovascular outcomes in patients with ST- elevation myocardial infarction who undergo primary percutaneous coronary intervention
Source: Int J Cardiol Cardiovasc Risk Prev. 2025 Mar 6;25:200383. doi: 10.1016/j.ijcrp.2025.200383 (PMC11951205; doi:10.1016/j.ijcrp.2025.200383)
Supplement: Multimedia component 1 [file mmc1.docx]

| **Met multiple exclusion criteria** |  |
| --- | --- |
| Use of immunosuppressant | 18 |
| Active infection | 7 |
| Active malignancy of haematological condition | 2 |
| Autoimmune condition | 1 |
| Eosinophilic condition | 17 |

(*Supplemental Table 1:* Reasons patients meeting multiple exclusions were excluded from the study.)

| **MACE** | **30 days, n (%)** | **1 year, (%)** |
| --- | --- | --- |
| All-cause mortality | 17 (4.6) | 23 (6.3) |
| Heart failure admission | 3 (0.8) | 8 (2.2) |
| Acute coronary syndrome | 11 (3.0) | 30 (8.2) |
| Cardiac related hospitalisation | 19 (5.2) | 54 (14.8) |
| Non fatal stroke | 3 (0.8) | 3 (0.8) |

(*Supplemental Table 2*: Number of patients (%) with a 30-day and 1-year MACE)

| *.* | **Analysis** | | | | |
| --- | --- | --- | --- | --- | --- |
|  | **Univariate** | | **Multivariate** | | |
| **ROC curve cut-off >-0.05** | **OR (95% CI)** | ***p*-value** | | **OR (95% CI)** | ***p*-value** |
| **MACE** |  |  | |  |  |
| 30-day | 3.489 (1.162-10.475) | 0.026 | | 3.266 (1.068-9.988) | 0.038 |
| 1 year | 1.629 (0.809-3.282) | 0.172 | | 1.501 (0.732-3.079) | 0.268 |
| **All-cause Mortality** |  |  | |  |  |
| 30-day | 8.7x10^7 (0.000-) | 0.997 | | 5.384x10^^7^ (0.000-) | 0.996 |
| 1 year | 3.300 (0.715-15.238) | 0.126 | | 2.790 (0.588-13.239) | 0.196 |

(*Supplement Table 3*: Univariate analysis of 30-day and 1-year MACE and all-cause mortality using the ROC curve cut-off of -0.05 as the dependent variable. Multivariate analysis of 30-day MACE with the ROC curve cut-off and other significant predictors of 30-day MACE)

|  | **Analysis** | | | | | | | | | |
| --- | --- | --- | --- | --- | --- | --- | --- | --- | --- | --- |
|  | **Univariate** | | | | **Multivariate** | | | | |  |
| **ROC curve cut-off >-0.05** | **OR (95% CI)** | ***p*-value** | | **OR (95% CI)** | | | ***p*-value** | |  |  |
| **Baseline Demographics** | | | | | | | | | |  |
| Age | 1.023 (0.997-1.049) | 0.081 | |  | | |  | |  |  |
| Male sex | 1.475 (0.729-2.946) | 0.271 | |  | | |  | |  |  |
| BMI | 0.965 (0.888-1.047) | 0.390 | |  | | |  | |  |  |
| **Medical comorbidities** | | | | | | | | | |  |
| Previous MI | 0.396 (0.624-3.295) | 1.434 | |  | | |  | |  |  |
| HTN | 1.239 (0.644-2.383) | 0.521 | |  | | |  | |  |  |
| Dyslipidaemia | 1.784 (0.926-3.439) | 0.084 | |  | | |  | |  |  |
| Smoker | 0.782 (0.399-1.532) | 0.473 | |  | | |  | |  |  |
| DM | 1.788 (0.878-3.639) | 0.109 | |  | | |  | |  |  |
| **Medication use** | | | | | | | | | |  |
| ACE | 1.246 (0.608-2.556) | 0.548 | |  | | |  | |  |  |
| Statin | 0.208 (0.772-3.288) | 1.593 | |  | | |  | |  |  |
| BB | 1.022 (0.341-3.057) | 0.969 | |  | | |  | |  |  |
| Aspirin | 1.147 (0.502-2.617) | 0.745 | |  | | |  | |  |  |
| Clopidogrel | 0.390 (0.051-2.985) | 0.364 | |  | | |  | |  |  |
| **Haematological parameters** | | | | | | | | | |  |
| Hb | 1.008 (0.989-1.028) | 0.394 | |  | | |  | |  |  |
| WCC | 1.047 (0.977-1.123) | 0.192 | |  | | |  | |  |  |
| Neutrophils | 1.002 (0.954-1.052) | 0.947 | |  | | |  | |  |  |
| Lymphocytes | 1.215 (0.994-1.486) | 0.058 | |  | | |  | |  |  |
| Monocytes | 3.372 (1.274-8.929) | 0.014 | | 1.793 (0.507-6.336) | | | 0.365 | |  |  |
| Baseline creatinine | 1.002 (0.997-1.008) | 0.421 | |  | | |  | |  |  |
| **Infarct characteristics** | | | | | | | | | |  |
| Peak troponin | 1 (1.000-1.000) | 0.113 | |  | | |  | |  |  |
| LAD culprit | 3.817 (1.879-7.754) | <0.001 | | 4.095 (1.630-10.289) | | | 0.003 | |  |  |
| Single-vessel disease | 0.795 (0.314-2.013) | 0.629 |  | | |  | |  |  |  |

(*Supplement Table 4*: Univariate and multivariate analysis of all predictors of 30-day MACE.)

|  | **Analysis** | | | |
| --- | --- | --- | --- | --- |
|  | **Univariate** | | **Multivariate** | |
| **ROC cut-off >-0.05** | **OR (95% CI)** | ***p*-value** | **OR (95% CI)** | ***p*-value** |
| **Baseline Demographics** |  |  |  |  |
| Age | 1.030 (1.010-1.051) | 0.003 | 1.008 (0.982-1.034) | 0.548 |
| Male sex | 1.376 (0.798-2.373) | 0.250 |  |  |
| BMI | 0.987 (0.927-1.050) | 0.678 |  |  |
| **Medical comorbidities** |  |  |  |  |
| Previous MI | 1.476 (0.767-2.841) | 0.244 |  |  |
| HTN | 1.063 (0.644-1.754) | 0.811 |  |  |
| Dyslipidaemia | 1.917 (1.150-3.193) | 0.013 | 1.459 (0.686-3.102) | 0.326 |
| Smoker | 0.681 (0.404-1.146) | 0.143 |  |  |
| DM | 2.167 (1.243-3.778) | 0.006 | 1.127 (0.492-2.585) | 0.777 |
| **Medication use** |  |  |  |  |
| ACE | 1.105 (0.627-1.948) | 0.729 |  |  |
| Statin | 1.592 (0.899-2.821) | 0.111 |  |  |
| BB | 1.799 (0.839-3.856) | 0.131 |  |  |
| Aspirin | 1.673 (0.913-3.064) | 0.096 |  |  |
| Clopidogrel | 0.870 (0.284-2.666) | 0.808 |  |  |
| **Haematological parameters** | |  |  |  |
| Hb | 0.995 (0.980-1.009) | 0.480 |  |  |
| WCC | 1.033 (0.975-1.094) | 0.269 |  |  |
| Neutrophils | 1.003 (0.967-1.041) | 0.860 |  |  |
| Lymphocytes | 0.995 (0.827-1.198) | 0.962 |  |  |
| Monocytes | 2.012 (0.907-4.459) | 0.085 |  |  |
| Baseline creatinine | 1.011 (1.003-1.019) | 0.010 | 1.007 (0.998-1.016) | 0.119 |
| **Infarct characteristics** |  |  |  |  |
| Peak troponin | 1.000 (1.000-1.000) | 0.160 |  |  |
| LAD culprit | 1.807 (1.091-2.991) | 0.021 | 2.072 (1.068-4.022) | 0.031 |
| Single-vessel disease | 1.542 (0.660-3.600) | 0.317 |  |  |

(*Supplement Table 5*: Univariate and multivariate analysis of all predictors of 1 year MACE.)

|  | **Analysis** | | | |
| --- | --- | --- | --- | --- |
|  | **Univariate** | | **Multivariate** | |
| **ROC cut-off >-0.05** | **OR (95% CI)** | ***p*-value** | **OR (95% CI)** | ***p*-value** |
| **Baseline Demographics** |  |  |  |  |
| Age | 1.045 (1.006-1.084) | 0.022 | 1.023 (0.963-1.087) | 0.458 |
| Male sex | 1.783 (0.671-4.739) | 0.246 |  |  |
| BMI | 0.921 (0.812-1.045) | 0.200 |  |  |
| **Medical comorbidities** |  |  |  |  |
| Previous MI | 2.292 (0.783-6.710) | 0.130 |  |  |
| HTN | 1.518 (0.575-4.007) | 0.399 |  |  |
| Dyslipidaemia | 2.000 (0.773-5.174) | 0.153 |  |  |
| Smoker | 1.417 (0.549-3.657) | 0.472 |  |  |
| DM | 2.397 (0.898-6.401) | 0.081 |  |  |
| **Medication use** |  |  |  |  |
| ACE | 1.939 (0.729-5.157) | 0.185 |  |  |
| Statin | 3.121 (1.188-8.197) | 0.021 | 2.285 (0.423-12.348) | 0.337 |
| BB | 1.189 (0.262-5.401) | 0.822 |  |  |
| Aspirin | 1.414 (0.446-4.484) | 0.556 |  |  |
| Clopidogrel | 0.959 (0.121-7.574) | 0.968 |  |  |
| **Haematological parameters** | |  |  |  |
| Hb | 0.996 (0.969-1.023) | 0.763 |  |  |
| WCC | 1.058 (0.966-1.159) | 0.223 |  |  |
| Neutrophils | 1.002 (0.935-1.074) | 0.959 |  |  |
| Lymphocytes | 1.299 (1.006-1.678) | 0.045 | 1.330 (0.847-2.089) | 0.216 |
| Monocytes | 5.777 (1.607-20.770) | 0.007 | 4.061 (0.515-32.013) | 0.183 |
| Baseline creatinine | 1.004 (0.998-1.010) | 0.153 |  |  |
| **Infarct characteristics** |  |  |  |  |
| Peak troponin | 1.000 (1.000-1.000) | 0.123 |  |  |
| LAD culprit | 5.138 (1.657-15.932) | 0.005 | 3.635 (0.665-19.868) | 0.136 |
| Single-vessel disease | 1.128 (0.251-5.076) | 0.875 |  |  |

(*Supplement Table 6*: Univariate and multivariate analysis of all predictors of 30-day all-cause mortality.)

|  | **Analysis** | | | |
| --- | --- | --- | --- | --- |
|  | **Univariate** | | **Multivariate** | |
| **ROC cut-off >-0.05** | **OR (95% CI)** | ***p*-value** | **OR (95% CI)** | ***p*-value** |
| **Baseline Demographics** |  |  |  |  |
| Age | 1.042 (1.008-1.007) | 0.016 | 1.029 (0.982-1.079) | 0.228 |
| Male sex | 1.820 (0.761-4.352) | 0.178 |  |  |
| BMI | 0.900 (0.800-1.012) | 0.078 |  |  |
| **Medical comorbidities** |  |  |  |  |
| Previous MI | 1.628 (0.578-4.583) | 0.356 |  |  |
| HTN | 1.511 (0.637-3.584) | 0.349 |  |  |
| Dyslipidaemia | 1.841 (0.788-4.301) | 0.158 |  |  |
| Smoker | 1.298 (0.557-3.023) | 0.546 |  |  |
| DM | 2.463 (1.024-5.921) | 0.044 | 2.670 (0.753-9.470) | 0.128 |
| **Medication use** |  |  |  |  |
| ACE | 1.982 (0.828-4.744) | 0.124 |  |  |
| Statin | 2.507 (1.042-6.030) | 0.040 | 1.013 (0.258-3.981) | 0.985 |
| BB | 0.892 (0.200-3.973) | 0.881 |  |  |
| Aspirin | 1.355 (0.481-3.814) | 0.565 |  |  |
| Clopidogrel | 1.614 (0.352-7.398) | 0.538 |  |  |
| **Haematological parameters** | |  |  |  |
| Hb | 0.986 (0.962-1.010) | 0.245 |  |  |
| WCC | 1.063 (0.979-1.154) | 0.145 |  |  |
| Neutrophils | 1.007 (0.956-1.062) | 0.789 |  |  |
| Lymphocytes | 1.226 (0.960-1.566) | 0.103 |  |  |
| Monocytes | 4.552 (1.400-14.796) | 0.012 | 2.126 (0.641-7.052) | 0.088 |
| Baseline creatinine | 1.005 (0.999-1.011) | 0.083 |  |  |
| **Infarct characteristics** |  |  |  |  |
| Peak troponin | 1.000 (1.000-1.000) | 0.086 |  |  |
| LAD culprit | 3.355 (1.345-8.367) | 0.009 | 2.126 (0.641-7.052) | 0.218 |
| Single-vessel disease | 1.505 (0.341-6.646) | 0.590 |  |  |

(*Supplement Table 7*: Univariate and multivariate analysis of all predictors of 1 year all-cause mortality.)
